# Global distribution of Xpert CT/NG assay *Neisseria gonorrhoeae* escape variants indicates sporadic emergence with limited clonality between 2016 and 2025

**DOI:** 10.2807/1560-7917.ES.2025.30.46.2500817

**Published:** 2025-11-20

**Authors:** Daniel Golparian, Maria Luiza Bazzo, Pamela Cristina Gaspar, Magnus Unemo

**Affiliations:** 1 WHO Collaborating Centre for Gonorrhoea and Other STIs, National Reference Laboratory for STIs, Department of Laboratory Medicine, Faculty of Medicine and Health, Örebro University, Örebro, Sweden; 2 Molecular Biology, Microbiology and Serology Laboratory, Federal University of Santa Catarina, Florianópolis, Brazil; 3 Department of HIV/AIDS, Tuberculosis, Viral Hepatitis, and Sexually Transmitted Infection, Secretariat of Health Surveillance and Environment, Ministry of Health of Brazil, Brasília, Brazil; 4 Institute for Global Health, University College London (UCL), London, United Kingdom

**Keywords:** *Neisseria gonorrhoeae*, gonorrhoea, GeneXpert, Xpert CT/NG, diagnostic-escape variant, multidrug-resistant *N. gonorrhoeae* (MDR-GC), antimicrobial resistance, travel

## Abstract

Screening of 54,837 gonococcal genomes identified 12 new variants lacking one (n = 9) or both (n = 3) of the Xpert CT/NG assay’s gonococcal targets. In total, 17 diagnostic-escape variants occurred across five countries and multiple genomic lineages; phylogenomic analysis revealed both ancestral and strain-specific recombination events. Xpert CT/NG diagnostic escape remains rare (0.026%) but illustrates recurrent recombination in gonococci. This emphasises the necessity of continuous external quality assessments, supplementary testing of gonococcal-positive molecular screening samples, and appropriate genomic and epidemiologic surveillance.

In June 2025, the United Kingdom (UK) Health Security Agency (UKHSA) reported a *Neisseria gonorrhoeae* strain that lacked the Cepheid Xpert CT/NG assay gonococcal targets (NG2 and NG4) following recombination with *Neisseria meningitidis*. A retrospective in silico screening revealed historical gonococcal isolates that lacked both targets (n = 1) or one of the targets (n = 3) [1]. Our aim was to assess the global distribution of Xpert CT/NG assay diagnostic-escape gonococcal variants and their spread across the global *N. gonorrhoeae* species phylogeny.

## Genomic in silico screening of Xpert CT/NG assay diagnostic-escape variants

**Figure 1:**
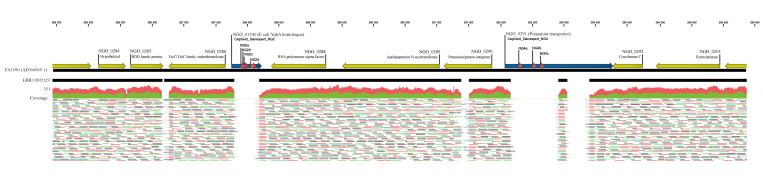
Location of the Xpert CT/NG assay gonococcal targets (NG2, NG4) and primer sequences mapped on the gonococcal FA1090 reference genome

We used the published Xpert CT/NG assay’s gonococcal target primer sequences from Cepheid’s United States (US) patent [2] to query 54,837 publicly available genomes of gonococcal isolates cultured in > 70 countries from 1928 to 2025 for the NG2 and NG4 targets. The NG2 and NG4 regions correspond to chromosomal loci NGO_01540 (GenBank AE004969.1 (287804–288188); UniProt A0A0H4IW08) and NGO_0291 (GenBank AE004969.1 (292050–293780); UniProt Q5F9U8), respectively, in the FA1090 reference genome (Figure 1). In silico PCR (https://github.com/simonrharris/in_silico_pcr) was performed using the published primer sequences [2] to detect the exact reference amplicons. A BLAST database was constructed from the reference amplicons, and each genome was queried with a mutation-tolerant search: 75% identity, 0% coverage threshold, ± 20 bp padding, and word size 7. Assemblies lacking a full-length hit for NG2 and/or NG4 were flagged as potential diagnostic-escape variants. Raw paired-end reads for 34 genomes suspected to lack NG2 and/or NG4 were mapped to FA1090, and manually inspected for deletions encompassing NG2 and NG4, as shown in Figure 1. Fourteen (0.026%) genomes from five countries, of which two genomes were previously found [1], among the 54,837 publicly available *N. gonorrhoeae* genomes from > 70 countries were confirmed to lack NG2 (n = 2), NG4 (n = 8), or both NG2 and NG4 (n = 4) (Table).

Mapping of paired-end Illumina reads to the *Neisseria gonorrhoeae* FA1090 reference genome showing the genomic region encompassing the NG2 and NG4 targets (blue arrows) of Cepheid’s Xpert CT/NG assay. Yellow arrows are adjacent loci. The red arrows are amplicon products corresponding to different primer sets described in the Cepheid’s United States patent [2], which define the NG2 and NG4 target loci used for diagnostic detection. Alternating red and green read pairs in the alignment track indicate forward and reverse sequencing orientations. The upper histogram shows total (red) and properly paired (green) read depth up to 351x.

**Table:** *Neisseria gonorrhoeae* isolates lacking Xpert CT/NG assay gonococcal targets (NG2, NG4) in the present study and a previous study [1], 5 countries, 2016–2025 (n = 17)

ENA accession	Study ID	Year	Country	MLST ST	NG-STAR ST	Lacking Xpert CT/NG target
ERR3578062^a^	PRJEB19989 [1]	2016	England	1596	4411	NG2, NG4
SRR8921865	PRJNA533242	2019	Canada	8156	442	NG2
SRR8922574	PRJNA533242	2019	Canada	8156	442	NG2
ERR11176299^a^	PRJEB58139 [1,13]	2020	France	10314	1615	NG4
ERR11892129	PRJEB62806	2020	Brazil	1596	4411	NG2, NG4
ERR11892399	PRJEB62806	2020	Brazil	1596	4336	NG2, NG4
SRR11724764	PRJNA317462	2020	US	13292	7845	NG4
SRR12612712	PRJNA317462	2020	US	11422	193	NG2, NG4
NA^a,b^	[1]	2020	England	10314	1615	NG4
NA^a,b^	[1]	2020	England	15679	165	NG2
SRR14109673	PRJNA317462	2021	US	9363	193	NG4
SRR14109675	PRJNA317462	2021	US	9363	193	NG4
SRR19127634	PRJNA317462	2022	US	9363	193	NG4
SRR23996852	PRJNA317462	2023	US	9363	193	NG4
SRR24565348	PRJNA317462	2023	US	9363	193	NG4
SRR26543840	PRJNA317462	2023	US	9363	193	NG4
NA^a,b^	[1]	2025	England	1596	4411	NG2, NG4

ENA: European Nucleotide Archive; ID: identification; MLST: multilocus sequence typing [14]; NA: not available; NG-STAR: *Neisseria gonorrhoeae* sequence typing for antimicrobial resistance [15]; ST: sequence type.

^a^ Previously reported by the United Kingdom Health Security Agency [1].

^b^ Three of the previously published isolates from England did not have any fastq files publicly available when the comprehensive search of 54,837 gonococcal genomes was performed.

## Confirmation of complete diagnostic escape using the Xpert CT/NG assay

At the World Health Organization (WHO) Collaborating Centre for Gonorrhoea and Other Sexually Transmitted Infections (STIs) (Örebro, Sweden), we had access to two (ERR11892129 and ERR11892399, cultured in 2020 in Brazil [3]), of the four isolates lacking both NG2 and NG4. Both isolates were tested in triplicate with the Xpert CT/NG assay on a GeneXpert platform (Cepheid). Neither target (NG2 or NG4) was detected in any of the triplicates of the isolates, which confirmed the in silico prediction of diagnostic escape. Notably, susceptibility testing using ETEST (BioMerieux) of the two Brazilian diagnostic-escape variants showed susceptibility to ceftriaxone, cefixime, azithromycin, ciprofloxacin and tetracycline.

## Phylogenomic context of Xpert CT/NG assay diagnostic-escape variants

**Figure 2:**
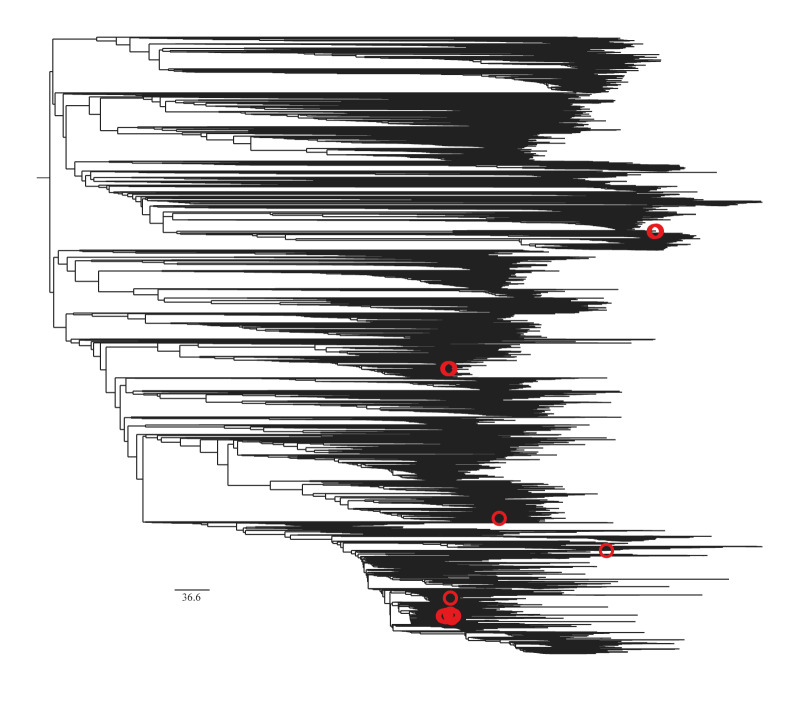
Phylogenomic tree of publicly available gonococcal genomes, > 70 countries, 1928–2025 (n = 54,837)

To assess genomic relatedness, the 14 genomes lacking NG2 and/or NG4 (with fastq files available) were identified in the global phylogenome of 54,837 *N. gonorrhoeae* genomes (Figure 2). The diagnostic-escape variants were dispersed across the phylogenomic tree, which indicates that recombinations resulting in the loss of the NG2 and/or NG4 targets in the Xpert CT/NG assay have occurred repeatedly and independently within the gonococcal population. Nevertheless, a small clade of isolates (n = 6), with identical genotypes from 2021 to 2023, lacking NG4 was observed from the US (Figure 2, Table). 

A neighbour-joining tree was generated in MBioSeq Ridom Typer using cgMLST allele profiles across 54,837 publicly available *Neisseria gonorrhoeae* genomes. Red rings denote isolates lacking one or both Xpert CT/NG assay’s gonococcal target loci (NG2 and/or NG4). The scattered placement of red-ringed genomes across different parts of the tree indicates that multiple independent recombination events have given rise to diagnostic-escape variants rather than a single monophyletic escape lineage. Notably, a small clade of isolates (n = 6), with identical genotypes from 2021 to 2023, lacking NG4 was observed from the United States.

**Figure 3:**
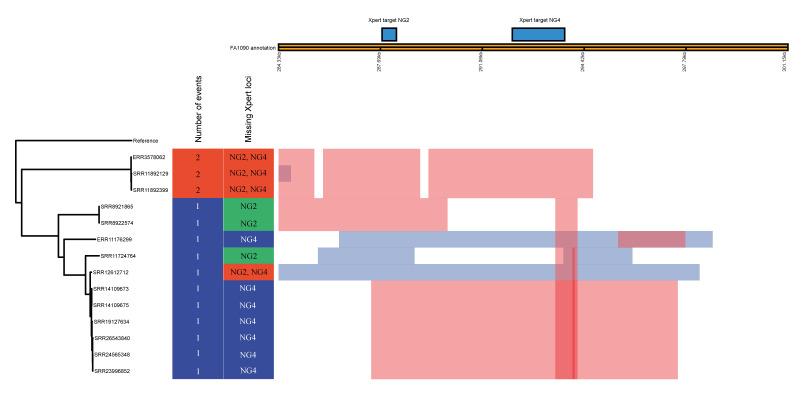
Recombination regions in gonococcal genomes lacking Xpert CT/NG assay gonococcal targets (NG2, NG4), 5 countries, 2016–2023 (n = 14)

Recombination mapping (Figure 3) further clarified distinct mechanisms that underlie the loss of the Xpert CT/NG assay NG2 and/or NG4 targets. Typically, two separate recombination events were required to eliminate both the NG2 and NG4 targets, resulting in a complete diagnostic escape; however, in some isolates, only a single localised mosaic fragment had replaced one of the target loci (typically NG4). Finally, a unique recombination was identified in genome SRR12612712 (Table), where a single large recombination event spanning both NG2 and NG4 occurring at the phylogenetic leaf, producing a complete diagnostic-escape variant in a single step. These patterns collectively demonstrate that recombination-mediated replacement of the Xpert CT/NG assay’s gonococcal target regions is a rare recurrence that can independently arise through different genomic pathways within *N. gonorrhoeae*.

Recombination regions identified by Gubbins [16] are shown as coloured horizontal blocks aligned to the FA1090 reference genome (brown bar) and ordered according to the phylogenetic tree. Each block represents a predicted recombination fragment within the affected taxa. Red blocks indicate ancestral recombination events that occurred in internal nodes of the phylogeny, whereas blue blocks correspond to strain-specific (happened at the leaf) recombination regions. The corresponding blocks illustrate regions where recombination has affected one or both loci. Together, these patterns highlight recurrent and independently acquired non-gonococcal *Neisseria* sequences encompassing the NG2–NG4 interval, including complete and partial replacements of the diagnostic target sites.

## Discussion

Our global survey demonstrates that *N. gonorrhoeae* strains lacking the Xpert CT/NG assay’s gonococcal targets (NG2 and/or NG4) remain extremely rare and are mostly genetically diverse. Their scattered occurrence across time and phylogeny argues against sustained transmission. Rather than emerging and expanding as a single ‘diagnostic-escape lineage’, these gonococcal variants have mainly evolved through independent divergent recombination events with non-gonococcal *Neisseria* strains, which is not rare in the gonococcal species with a high natural competence for DNA exchange [4]. However, a minor clade of isolates lacking the NG4 target because of a single recombination event was observed in the US from 2021 to 2023, and should be closely monitored in future genomic studies. Fortunately, the NG2 target was intact in these isolates and will be detected accordingly by the Xpert CT/NG assay. Nevertheless, the Xpert CT/NG assay requires both targets (NG2 and NG4) to be detected to report a positive gonococcal result. Thus, for the gonococcal variants lacking one of these targets (NG2 or NG4), the PCR curves should be evaluated and supplementary testing performed. It would be valuable if the software of the Xpert CT/NG assay automatically reported that supplementary testing with a nucleic acid amplification test (NAAT) targeting another gonococcal sequence(s) should be performed in these cases. Additionally, to be able to monitor the evolution of the target sequences in all diagnostic gonococcal NAATs, these sequences should be publicly available. Notably, all gonococcal variants lacking NG2 and/or NG4 were identified in 2016–2025. This may reflect that the vast majority of publicly available genomes were sequenced during the recent decade; however, a diagnostically selected evolution by frequent use of the Xpert CT/NG assay cannot be completely excluded.

Recombination analysis showed diagnostic-escape variants arose from strain-specific events, but several recombination fragments were ancestral and shared among multiple related isolates, indicating limited clonal expansion. This suggests that, although the phenomenon is currently sporadic, future expansion of an ancestral recombinant lineage could increase the clinical relevance of diagnostic-escape variants.

Gonococcal variants escaping detection in many commercial and in house molecular diagnostic assays have previously been transmitted internationally [5,6]. Additionally, occasional *N. meningitidis* variants, which evolved through recombination with gonococcal strains and resulted in false-positive results in several commercial gonococcal NAATs, have been described [7-9]. The results of the present study and previous studies describing the loss of one or two diagnostic targets or false-positive results in diagnostic gonococcal NAATs [1,5-9] emphasise the importance of supplementary molecular testing (using a NAAT with another gonococcal target sequence(s)) of samples with anomalous or discordant results and all gonococcal screening-positive samples, as recommended in the European Gonorrhoea guideline [10]. To additionally improve the antimicrobial resistance surveillance, it is recommended that gonococcal culture is attempted from patients with gonococcal-positive results in diagnostic NAATs, where feasible and especially for symptomatic patients, which will also further confirm the results of the screening NAAT. Furthermore, regular evaluations of different diagnostic NAATs are recommended, as well as frequent participation in appropriate international external quality assessments (EQAs) that should include currently circulating gonococcal strains as well as temporally, geographically and genetically diverse strains (including variants causing false-negative and false-positive NAAT results) and NAATs targeting different sequences. For this, it is imperative that gonococcal variants escaping detection or variants of other pathogens causing false-positive results in different NAATs are shared with national and international reference laboratories and EQA panel providers. Finally, routine genomic surveillance should be closely integrated with diagnostic performance monitoring and antimicrobial resistance surveillance, to monitor the evolution of the NAAT target sequences and ensure early detection and mitigation of variants resulting in false-negative or false-positive results in diagnostic NAATs, as well as detection of multidrug-resistant or extensively drug-resistant gonococcal strains [11,12]. The integration of genomic surveillance will also contribute to enhanced understanding on how *N. gonorrhoeae* is evolving including the emergence, clonality, and fitness advantages/disadvantages of gonococcal variants escaping detection or other pathogens causing false-positive results in different diagnostic NAATs.

## Conclusion

The global prevalence of diagnostic-escape variants for the Xpert CT/NG assay is low and no evidence of widespread expansion or transmission was observed. However, laboratories and clinics should remain alert for unusual diagnostic results and potentially false-negative or false-positive results when using the Xpert CT/NG assay and other diagnostic gonococcal NAATs. Our findings underscore that diagnostic escape is not an isolated event but a recurrent evolutionary process that will continue to happen within the naturally recombinogenic gonococcal species. This underscores the importance of frequent participation in appropriate EQAs, continuous supplementary testing of gonococcal-positive NAAT samples [10], and routine genomic and epidemiologic surveillance, to early detect false-negative or false-positive gonococcal variants and preserve the reliability of molecular diagnostic assays for gonorrhoea.

## Data Availability

The first author (DG) and the last author (MU) had full access to all the data in the study and datasets can be made available by the corresponding author after publication on reasonable request.
